# Comprehension of Co-Speech Gestures in Aphasic Patients: An Eye Movement Study

**DOI:** 10.1371/journal.pone.0146583

**Published:** 2016-01-06

**Authors:** Noëmi Eggenberger, Basil C. Preisig, Rahel Schumacher, Simone Hopfner, Tim Vanbellingen, Thomas Nyffeler, Klemens Gutbrod, Jean-Marie Annoni, Stephan Bohlhalter, Dario Cazzoli, René M. Müri

**Affiliations:** 1 Perception and Eye Movement Laboratory, Departments of Neurology and Clinical Research, Inselspital, University Hospital Bern, and University of Bern, Bern, Switzerland; 2 Division of Cognitive and Restorative Neurology, Department of Neurology, Inselspital, Bern University Hospital, and University of Bern, Bern, Switzerland; 3 Neurology and Neurorehabilitation Center, Department of Internal Medicine, Luzerner Kantonsspital, Luzern, Switzerland; 4 Neurology Unit, Laboratory for Cognitive and Neurological Sciences, Department of Medicine, Faculty of Science, University of Fribourg, Fribourg, Switzerland; 5 Gerontechnology and Rehabilitation Group, University of Bern, Bern, Switzerland; University of Barcelona, SPAIN

## Abstract

**Background:**

Co-speech gestures are omnipresent and a crucial element of human interaction by facilitating language comprehension. However, it is unclear whether gestures also support language comprehension in aphasic patients. Using visual exploration behavior analysis, the present study aimed to investigate the influence of congruence between speech and co-speech gestures on comprehension in terms of accuracy in a decision task.

**Method:**

Twenty aphasic patients and 30 healthy controls watched videos in which speech was either combined with meaningless (baseline condition), congruent, or incongruent gestures. Comprehension was assessed with a decision task, while remote eye-tracking allowed analysis of visual exploration.

**Results:**

In aphasic patients, the incongruent condition resulted in a significant decrease of accuracy, while the congruent condition led to a significant increase in accuracy compared to baseline accuracy. In the control group, the incongruent condition resulted in a decrease in accuracy, while the congruent condition did not significantly increase the accuracy. Visual exploration analysis showed that patients fixated significantly less on the face and tended to fixate more on the gesturing hands compared to controls.

**Conclusion:**

Co-speech gestures play an important role for aphasic patients as they modulate comprehension. Incongruent gestures evoke significant interference and deteriorate patients’ comprehension. In contrast, congruent gestures enhance comprehension in aphasic patients, which might be valuable for clinical and therapeutic purposes.

## Introduction

Human communication consists of both verbal (speech) and nonverbal (facial expressions, hand gestures, body posture, etc.) elements. Gesturing is a crucial part of human nonverbal communication and includes co-speech gestures—communicative movements of hands and arms that accompany concurrent speech [1–3]. After a left-hemispheric stroke, patients often develop aphasia, defined as the acquired loss or impairment of language [4]. Impairments in verbal elements of language processing in aphasia are well known and extensively studied (e.g., [4, 5]). However, less is known about potential mechanisms and impairments in non-verbal aspects and, in particular, it is uncertain to what extent gesturing influences comprehension in aphasia.

There is evidence that gesturing may be preserved in aphasic patients [6–8], either facilitating speech processing (e.g., [9, 10]) or compensating for its impairment [6, 11]. This has led to the theoretical assumption that speech and gesturing depend on two independent cortical systems [10, 12, 13]. However, other aphasic patients have considerable problems to produce or understand gestures [3, 14–17]. Further research on gesture processing in aphasia can contribute to the ongoing debate of whether gesturing and speech rely on two independent cortical systems (with the implication that gestures could substitute or facilitate impaired speech), or whether they are organized in overlapping systems of language and action (e.g., [18–20]). Studying the perception of co-speech gestures in aphasia is thus relevant for two more reasons. First, aphasia can be considered as a disorder with supra-modal aspects [4]. Thus, it seems important to gain insights into the mechanisms leading to impairment of not only verbal aspects, but also of nonverbal ones, such as gesture perception and processing. Second, understanding the role of gestures in language comprehension in aphasic patients is also of clinical relevance. Research in this field may lead to new therapeutic approaches, e.g., the development of compensatory strategies for impaired verbal communication in aphasic patients, for instance during the activities of daily living.

Only few studies (e.g., [21, 22]) examined perception of co-speech gestures in aphasic patients. Previous research has mostly concentrated on comprehension of pantomime gestures (i.e. imitation of actions by means of gestures produced in the absence of speech). To the best of our knowledge, only two studies investigated speech and gesturing integration in aphasic patients. In one of these studies, Records [23] presented information either auditory (target word), visually (referential gesture towards target picture), or as a combination of both modalities (target word and referential gesture). Furthermore, the authors varied the level of ambiguity of the input. Aphasic patients had to indicate in a forced-choice task which picture had been described. The authors found that when auditory and visual information were ambiguous, aphasic patients relied more on the visually presented referential gesture [23]. More recently, in a single case study with a similar forced-choice paradigm, Cocks, Sautin, Kita, Morgan, and Zlotowitz [24] showed video vignettes of co-speech gestures to an aphasic patient and to a group of healthy controls. All participants were asked to select among four alternatives (including a verbal and a gestural match) the picture corresponding to the vignette they had watched. In order to solve the task, the aphasic patient relied primarily on gestural information. In contrast, healthy controls relied more on speech information [24]. The paradigm applied by Cocks and colleagues [24] allowed to assess another important aspect of co-speech gestures, namely the phenomenon of multimodal gain. This phenomenon refers to the fact that the integration of two modalities (here gesturing and speech) leads to better performance than one of the two modalities alone, as often observed in healthy participants (e.g., [25–30]; for a review see [31]). Cocks et al.’s results showed that this integration phenomenon was impaired in their aphasic patient, who showed a lower multimodal gain than healthy controls [24]. However, due to the single case nature of the study, it remains unclear whether this impairment can be generalized to all aphasic patients.

When studying speech and gesturing in aphasic patients, the frequent co-occurrence of limb apraxia (i.e., a higher cognitive impairment of motor control and conduction of movements [32, 33]), has to be taken into account. Lesions to left-hemispheric temporo-frontal areas often lead to both language impairment and apraxia (e.g., [15, 18, 34]). This co-occurrence is due to the large overlap of the cortical representation of language, limb praxis, and higher-order motor control. It is assumed [32] that apraxia influences not only gesture production, but also gesture comprehension. The influence of apraxia on gesture comprehension has been investigated by several studies (e.g., [15, 35–38]), but yielded controversial results. Halsband et al. [36] found impaired gesture imitation in apraxic patients, but no clear influence on gesture comprehension. In contrast, Pazzaglia et al. [35] reported a strong correlation between the performance in gesture imitation and gesture comprehension. The same group [38] found also gesture comprehension deficits in patients with limb apraxia. In a later study, they reported a specific deficit in gesture discrimination in a sample of patients with primary progressive aphasia [37]. Apraxia-related deficits may further complicate communicative attempts in aphasic patients [34]. In order to develop targeted speech-language therapy approaches, it may therefore be valuable to know which patients would benefit from additional, tailored gesture-based therapy.

Eye movement tracking has grown in importance in the field of cognitive neuroscience over the last few decades. Eye-tracking is a highly suitable method to measure fixation behavior, and to assess visual perception and attention to gestures (e.g., fixations on a moving / gesturing hand) or to speech (e.g., fixations on a speaker’s lip movements) ([39]; for a review see also [40]). Eye-tracking techniques have been used for the study of gestures and speech-related behavior (e.g., [39, 41–43]). These investigations have shown that healthy participants spend as much as 90–95% of the fixation time on the speaker’s face in live conditions, and about 88% in video conditions. Only a minority of fixations is directed towards gestures [39, 42, 43]. Several factors are supposed to influence visual exploration behavior in healthy participants, such as the gestural amplitude and gestural holds throughout the execution of the gesture, the direction of the speaker’s own gaze, and differences in gestural categories [39, 42]. However, it is unclear whether aphasic patients display similar fixation patterns. To date, there do not appear to have been any studies investigating the visual exploration behavior during the observation of congruent or incongruent co-speech gestures.

The present study aimed to investigate two main research questions in a sample of aphasic patients in comparison to healthy controls. First, we aimed to assess the influence of congruence between speech and co-speech gestures on the comprehension of speech and gestures in terms of accuracy in a decision task. Second, we were interested how the perception, i.e., the visual exploration behavior, is influenced by different levels of congruence.

To assess these questions, we created an experiment comprising short video sequences with varying levels of congruence between speech and co-speech gestures. Each video consisted of a simple spoken sentence that was accompanied by a co-speech gesture. During the presentation of the videos, infrared-based eye-tracking was used to measure visual exploration on the hands and the face of the speaker. Three conditions of varying congruence were tested: a baseline condition (i.e., speech combined with a meaningless gesture), a congruent condition (i.e., speech and gesture having the same meaning), and an incongruent condition (i.e., speech combined with a non-matching, but semantically meaningful, gesture). After the presentation of each video, the participants had to decide whether the spoken sentence was congruent with respect to the gesture (yes/no answer, forced-choice). Accuracy in the forced-choice task and visual exploration were assessed in a group of aphasic patients, and compared to those of a group of age- and gender-matched healthy participants, who underwent the same procedure.

Concerning our first aim and in accordance with previous reports (e.g., [4, 44–48]), we assume that aphasic patients generally display specific language processing (i.e., comprehension) deficits. We thus assume a priori that aphasic patients perform less accurately compared to healthy controls in the baseline condition, where meaningless gestural stimuli provide neither additional information nor semantic interference. Our first hypothesis on the influence of congruence between speech and co-speech gestures is based on previous findings showing that co-speech gestures facilitate language comprehension in healthy participants, by providing additional or even redundant semantic information (e.g., [25–29]; for a review see [31]). We thus hypothesize that congruent co-speech gestures will have a facilitating effect on comprehension, due to the presentation of additional congruent information. In contrast, incongruent gestures should result in reduced comprehension, due to the interference of the conflicting semantic contents of speech and co-speech gesture.

Furthermore, we were interested in the role of apraxia. If apraxia plays an important role on comprehension of speech and co-speech gestures, then we expect that the comprehension in aphasic patients would not be influenced by different conditions of congruence, since the patients would have no additional gain of the co-speech gesture information. We thus hypothesize that both aphasia and apraxia severity interfere with the comprehension of speech and gesturing, however, this interference could be differentially strong depending on patients’ specific impairments as well as other cognitive deficits. In an additional control experiment, we tested comprehension of isolated gestures, evaluating the possibility that comprehension of gestures per se would be impaired.

The second aim was to analyze visual exploration behavior during performance of the task and evaluate different exploration strategies between patients and healthy controls. We assume that both healthy controls and patients would fixate the face region the most, as shown by previous reports [39, 42, 43]. Due to the design of our study, where gestures play a prominent role, we hypothesize nevertheless a larger amount of fixations on the hands than previously reported. Furthermore, we hypothesize differences in visual exploration between aphasic patients and healthy controls: due to the impaired language comprehension in aphasia, patients may not use verbal information as efficiently as healthy controls. If aphasic patients rely more on nonverbal information, such as co-speech gestures, then they should look more at the gesturing hands. This would result in increased fixation durations on the hands and decreased fixation durations on the face, compared to healthy controls. However, if apraxia has a stronger impact on visual exploration behavior than the language-related deficits (i.e., gestures become less comprehensible and less informative for aphasic patients with apraxia), then we may find decreased fixation durations on co-speech gestures and increased fixation durations on the face in comparison to healthy controls. Taken together, we were hypothesizing that aphasia and apraxia severity could differentially interfere with comprehension and the influence of congruence between speech and gesturing on such comprehension.

## Materials and Method

### Declaration of ethical approval

All participants gave written informed consent prior to participation. Ethical approval to conduct this study was provided by the Ethical Committee of the State of Bern. The study was conducted in accordance with the principles of the latest version of the Declaration of Helsinki. The individual in this manuscript has given written informed consent (as outlined in PLOS consent form) to publish these case details.

### 2.1 Participants

Twenty patients with aphasia after a left-hemispheric stroke in cortical-subcortical regions (13 men, age: *M* = 56.7, *SD* = 13.5) and 30 age- and gender-matched healthy controls (14 men, age: *M* = 51.9, *SD* = 17.8) participated in the study. There was no significant difference between the two groups with respect to age (*t*(48) = 1.19; *p* = .23) or gender ratio (χ2(1) = 1.62; *p* = .25). All participants were right-handed. The native language of all participants was German. Aphasic patients were recruited from three different neurorehabilitation clinics in the German speaking part of Switzerland (University Hospital Bern, Kantonsspital Luzern, and Spitalzentrum Biel). At the time of examination, aphasic patients were in a sub-acute to chronic state (i.e., 1.5 to 55 months post stroke onset, *M* = 14.4, *SD* = 16.4). Aphasia diagnosis and classification was based on neurological examination and on standardized diagnostic language tests, administered by experienced speech-language therapists. Diagnostic measurements were carried out within two weeks of participation in the study. To assess aphasia severity and classify aphasia type, two subtests of the Aachener Aphasie Test (AAT, [49]) were carried out, i.e., the Token Test and the Written Language Test. The AAT is a standardized, well-established diagnostic aphasia test battery for German native speakers. Willmes, Poeck, Weniger and Huber [50] showed that the discriminative validity of the two selected subtests (i.e., Token Test and Written Language) is as good as the discriminative validity of the full test battery. In addition, the Test of Upper Limb Apraxia (TULIA, [51]) was administered to assess limb apraxia. The TULIA is a recently developed test, which consists of 48 items divided in two subscales (imitation of the experimenter demonstrating a gesture, and pantomime upon verbal command, respectively) with 24 items each. Each subscale consists of 8 non-symbolic (meaningless), 8 intransitive (communicative), and 8 transitive (tool related) gestures. Rating is preferably performed by means of offline video analysis, on a 6-point rating scale (0–5), resulting in a score range of 0–240. Offline video-based rating yields good to excellent internal consistency, as well as test-retest-reliability and construct validity [51]. Twelve out of the 20 aphasic patients were additionally diagnosed with apraxia according to the cut-off score defined by the TULIA test. Patients’ demographic and clinical data are summarized in Tables 1 and 2. All participants had normal or corrected-to-normal visual acuity and hearing, and no history of psychiatric disorders. Patients with complete hemianopia involving the fovea or right-sided visual neglect were excluded from the study.

**Table 1:** Overview of demographic and clinical data of aphasic patients and controls.

		Patients	Controls
		*n* = 20	*n* = 30
Age	Mean	56.7	51.9
(in years)	Range	34–75	19.83
Gender	Male	13	14
	Female	7	16
Months post-onset	Mean	14.4	
	SD	16.4	
Number of errors in the Token Test	Mean	18.6	
(max. 50, cut-off > 7)	SD	16.5	
	Range	0–50	
Number of correct items in the Written Language	Mean	56.2	
(max. 90, cut-off < 81)	SD	28.4	
	range	0–86	
Number of correct items in the TULIA	Mean	188.1	
(max. 240, cut-off < 194)	SD	21.5	
	range	141–221	
Number of correct items in the TULIA Imitation Subscale	Mean	94.7	
(max. 120, cut-off < 95)	SD	11.8	
	range	71–110	

*Notes*. SD = Standard Deviation; Token Test: age-corrected error scores; Written Language: raw scores; TULIA = test of upper limb apraxia.

**Table 2:** Detailed demographic and clinical data of aphasic patients.

									AAT		TULIA	
Patient Number	Gender	Age	Years of Education	Etiology	Lesion Location	Months post-onset	Presence of Hemiparesis	Aphasic Syndrome Type	Token TestScore	Written LanguageScore	OverallScore	Imitation Subscale Score
1	M	61	14	isch	L temp/par	3.3	no	amnestic	20	67	204	99
2	F	53	16	isch	L temp/par	4.5	no	amnestic	0	n/a	221	106
3	M	74	15	isch	L front/temp	19.3	no	Broca	0	80	201	97
4	F	51	12	isch	L front/temp	1.7	no	Broca	18	41	171	97
5	F	40	17	hem	L front/par	4.0	yes	Broca	50	n/a	159	79
6	F	66	12	isch	L temp	1.6	no	amnestic	8	53	206	100
7	F	46	12	isch	L front/temp	41.6	no	Broca	7	60	212	105
8	M	71	12	isch	L temp/par	2.0	no	Broca	50	n/a	156	71
9	M	73	14	isch	L temp	1.5	no	Wernicke	17	81	168	81
10	F	40	17	isch	L temp/par	4.6	no	amnestic	19	80	207	110
11	M	69	12	isch	L temp	4.7	no	Broca	0	70	216	104
12	F	36	17	hem	L front/temp/par	55.0	yes	global	39	23	186	94
13	M	47	13	isch	L front/temp/par	36.0	no	Broca	11	75	192	97
14	M	34	11	vasc	L front/temp/par	13.3	yes	global	50	0	141	74
15	M	56	12	isch	L temp/par	37.5	no	Broca	11	n/a	189	94
16	M	67	13	isch	L temp/par	30.0	no	Wernicke	27	86	195	107
17	M	75	12	isch	L temp	8.7	no	Wernicke	0	60	168	80
18	M	62	14	isch	L temp/par	6.0	no	Wernicke	6	69	188	91
19	M	70	12	hem	L temp/par	10.7	no	Wernicke	7	67	192	100
20	M	42	12	isch	bilateral	2.0	no	Wernicke	13	79	189	108

*Notes*. L = left; Etiology: isch = ischaemic infarction in the territory of the medial cerebral artery, hem = hemorrhagic infarction (parenchymal hemorrhage), vasc = vasculitis; Lesion Location: front = frontal, par = parietal, temp = temporal; AAT = Aachener Aphasie Test, Token Test: age-corrected error scores; Written Language: raw scores; n/a: not applicable; TULIA = test of upper limb apraxia.

### 2.2 Lesion Characteristics

**Fig 1:**
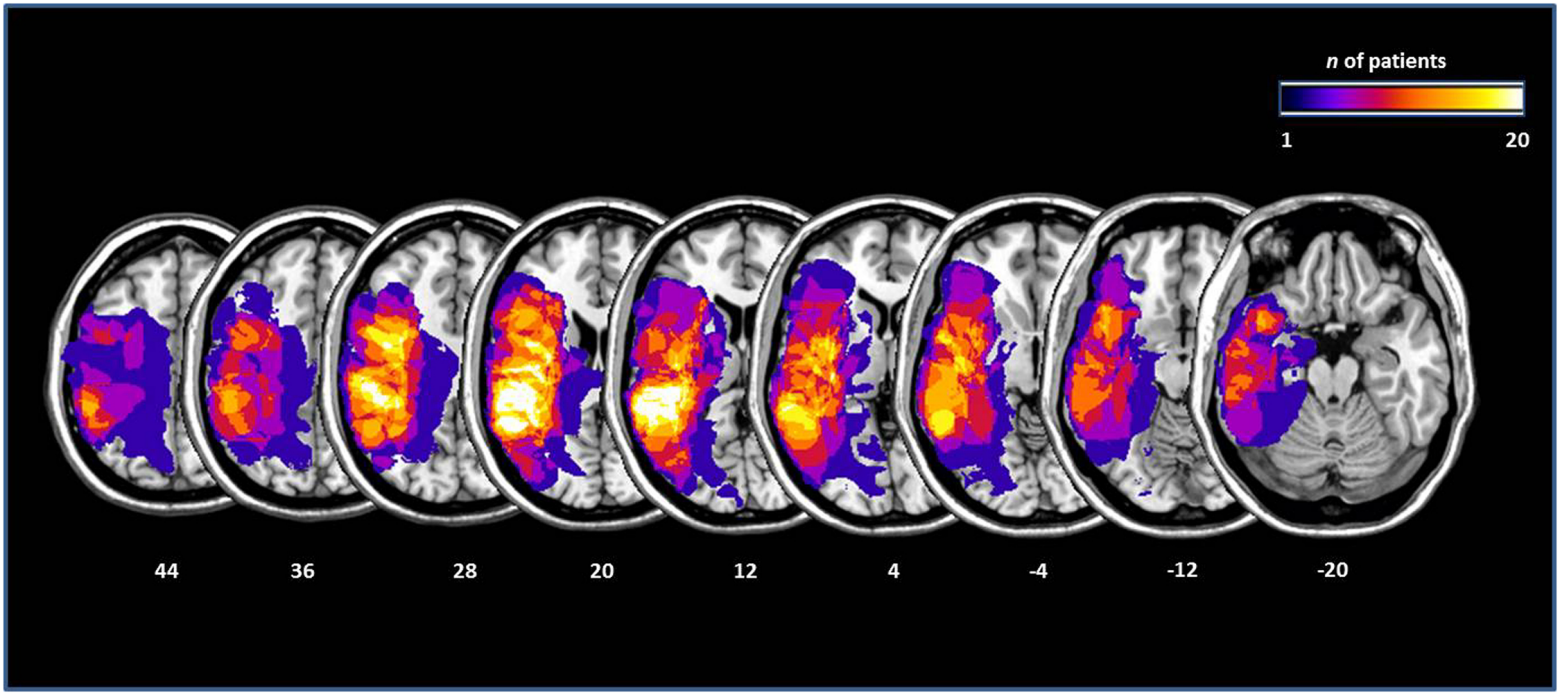
Lesions maps of the 20 aphasic patients, plotted on axial slices oriented according to the radiological convention. Slices are depicted in 8mm descending steps. The *Z* position of each axial slice in the Talairach stereotaxic space is presented at the bottom of the figure. The number of patients with damage involving a specific region is color-coded according to the legend.

Lesion mapping was performed by a collaborator who was naïve with respect to the patients’ test results and clinical presentation. An independent, second collaborator checked the accuracy of the mapping. Lesion mapping was performed using the MRIcron software [52]. We used the same procedure as applied by Karnath et al. [53, 54]. Diffusion-weighted scans were selected for the analysis when MRI sequences were obtained within the first 48 h post-stroke. Magnetic resonance imaging (MRI) scans were available for 13 patients, and computed tomography (CT) scans were available for the remaining seven patients. For the available MRI scans, the boundary of the lesions was delineated directly on the individual MRI images for every single transversal slice. Both the scan and the lesion shape were then mapped into approximate Talairach space using the spatial normalization algorithm provided by SPM5 (http://www.fil.ion.ucl.ac.uk/spm/). For CT scans, lesions were mapped directly on the T1-weighted MNI single subject template implemented in MRIcron [55] and visually controlled for different slice angles. The mean lesion volume was 56.7cm^3^ (SEM = 13.56cm^3^). Fig 1 shows the localisation and the degree of overlap of the brain lesions, transferred to the standard ch2 brain template implemented in MRICron ([55]).

### 2.3 Stimulus Material

**Fig 2:**
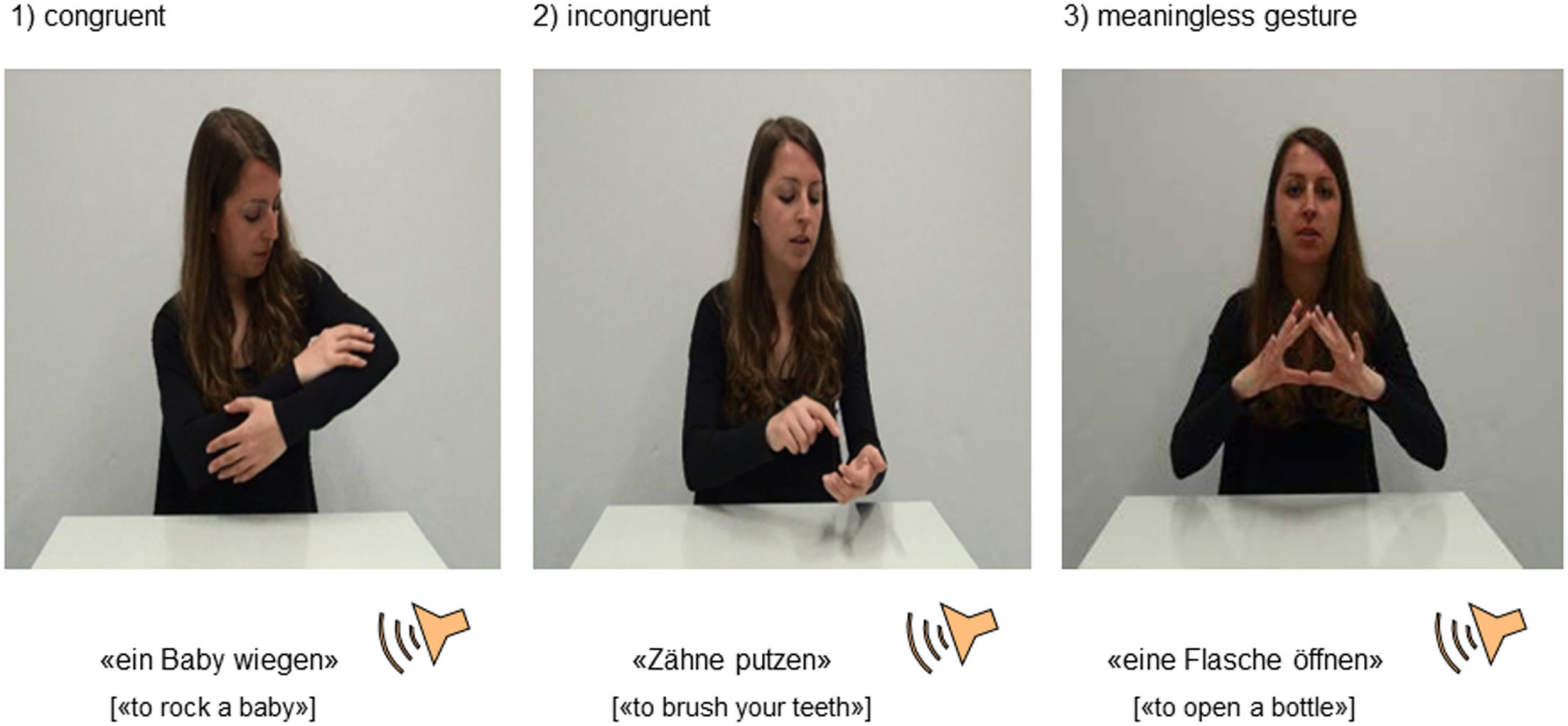
Examples of the video sequences used as stimuli, each consisting of simultaneous speech and gesturing. The sequences were either congruent (1), incongruent (2), or speech was combined with a meaningless gesture (3).

Three experimental conditions were implemented; each consisting of different short video sequences (Fig 2). In the first condition, the meaningless condition serving as a baseline, speech was simultaneously combined with meaningless gesturing (e.g., an actress saying “to open a bottle” and simultaneously putting her fingertips together). In the second condition, the congruent condition, sequences contained simultaneous speech and gesturing with matching content (e.g., an actress saying “to rock a baby” and simultaneously mimicking the same action, i.e., joining her hands in front of her torso, with the arms forming an oval shape, as if holding a baby, and performing an oscillating movement with her hands and arms). In the third condition, the incongruent condition, sequences contained simultaneous speech and gesturing with non-matching content (e.g., an actress saying “to brush your teeth” and simultaneously mimicking the action of dialing a number on a phone, hence creating incongruence between speech and gesturing). Most of the videos (47 out of 75) depicted actual motor actions, while 28 videos were symbolic actions (e.g., saying “it was so delicious” while showing a thumbs-up gesture of approval). Each video sequence was followed by a forced-choice task, in which participants were prompted to decide by key press whether speech and gesturing were congruent or not. Congruent trials were correctly answered by pressing the “yes”-key, whereas both the meaningless and the incongruent trials were correctly answered by pressing the “no”-key. We therefore decided to include more trials in the congruent condition. Out of the total 75 videos, 33 were congruent, 25 were incongruent, and 17 were meaningless. A list of the content of the original stimuli in German, as well as their English translation, can be found in S1 Appendix.

### 2.4 Apparatus and Eye-Tracking

Eye movements were measured by means of a remote RED eye-tracking system (RED 250, SensoMotoric Instruments GmbH, Teltow, Germany), attached directly under the screen used for stimulus presentation. This infrared-based system allows the contactless measurement of the eye movements, of the number of visual fixations on specific regions of interest (ROIs), of the cumulative or mean fixation duration, and of the percentage gaze time on specific ROIs. A major advantage of the RED eye-tracking system is that fixation or stabilization of the head is not necessary, since the system is equipped with an automatic head-movement compensation mechanism (within a range of 40 x 20 cm, at approximately 70 cm viewing distance). The system was set at 60 Hz sampling rate (temporal resolution).

### 2.5 Procedure

Participants were seated on a chair, at a distance varying between 60 and 80cm, facing the 22” computer screen where the videos were presented. A standard keyboard was placed in front of the participants at a comfortable distance. Participants were asked to carefully watch the video sequences and listen to the simultaneously presented speech. Moreover, they were instructed to decide, after each sequence, whether speech and gesturing had been congruent or incongruent. For this purpose, a static question slide appeared after each sequence. Participants had to enter their response by pressing one out of two keys on a standard keyboard within 6 seconds. The answer keys were color-coded, i.e., a green sticker indicating “yes” (covering the X-key of the keyboard), and a red sticker indicating “no” (covering the M-key of the keyboard). No additional verbal instruction was given. Three practice trials (one for each condition, i.e., congruent, incongruent, and baseline) were administered prior to the main experiment. During practice, feedback was given to the participants. Erroneous trials were explained and repeated to enhance task comprehension.

In the main experiment, the 75 video sequences were presented in randomized order. Four short breaks were included in the design in order to avoid fatigue, resulting in five blocks of 15 random sequences each. Before each block, a 9-point calibration procedure was performed, in order to ensure accurate tracking of participants’ gaze. During calibration, participants were requested to fixate as accurately as possible 9 points, appearing sequentially and one at a time on the screen. The quality of the calibration was assessed by the experimenter, aiming for a gaze accuracy of 1° visual angle on the x- and y-coordinates or better. If this criterion was not met, the calibration procedure was repeated.

To assess participants’ comprehension of isolated gestures, we performed an additional control experiment. The aim of this experiment was to exclude the possibility that gesture comprehension per se was impaired, which in turn might have influenced comprehension in combined conditions (i.e., speech and gesturing). In this control experiment, participants were presented with a block of 15 video sequences in randomized order. The video sequences contained gestures without any verbal utterance. Participants were asked to carefully watch the gestures. After each video sequence, they were asked to indicate the meaning of the presented gesture by means of a forced-choice task. Three possible definitions of each gesture were presented, i.e. the correct definition, a semantic distractor, and a phonological distractor.

### 2.6 Data Processing and Analysis

#### 2.6.1 Accuracy of speech and co-speech gesture comprehension

For every video sequence, an accuracy rate was calculated within the group of healthy participants (i.e., the percentage of healthy participants answering correctly to that particular video sequence). Two out of the 75 video sequences (a congruent and a meaningless one, respectively) were excluded from further analysis, because healthy participants’ accuracy rate in these sequences was below -1 standard deviation with respect to the mean of the accuracy rates of all sequences. The percentage of correct responses was calculated for each participant in the remaining 73 video sequences. Data were analyzed by means of a repeated-measures ANOVA with the between-subjects factor Group (levels: patients, controls) and the within-subjects factor Condition (levels: congruent, incongruent, meaningless). In order to eliminate overall gesture comprehension as a confounding factor, an ANCOVA with the covariate gesture comprehension (accuracy in the control experiment) was calculated.

All statistical analyses were carried out using SPSS 21.0 and STATISTICA 6.0.

#### 2.6.2 Visual Exploration

The visual exploration behavior of the participants was evaluated by analyzing the cumulative fixation duration on two predefined ROIs of the video sequences, namely the face and the gesturing hand(s) of the actress. Visual fixation data were preprocessed with the BeGaze^™^ analysis software (SensoMotoric Instruments GmbH, Teltow, Germany). Visual fixations from participants’ right eye were analyzed, since all participants had conjugate eye movements [56]. Fixations shorter than 100ms were excluded from further analysis, as it is not clear whether information processing takes place during visual fixations shorter than this cut-off [57, 58]. For each video sequence, the cumulative fixation duration was computed over the predefined ROIs. The duration of all fixations on the predefined ROIs was summed (in milliseconds) and weighted (to balance the uneven number of stimuli between the three experimental conditions).

Two out of the 20 aphasic patients had to be excluded from eye-tracking analyses due to low tracking ratios (more than 1 standard deviation lower than the mean of the whole group) of their eye movements. Cumulative fixation duration data were analyzed by means of a repeated-measures ANOVA with the between-subjects factor Group (levels: patients, controls) and the within-subjects factors Condition (levels: congruent, incongruent, meaningless), and ROI (levels: face, gesturing hand(s)). For the control experiment, an analogous repeated-measures ANOVA was computed on accuracy and cumulative fixation duration, with the between-subjects factor Group (levels: patients, controls) and the within-subjects factor ROI (levels: face, gesturing hand(s)).

For all repeated-measures ANOVAs, Greenhouse-Geisser corrections were applied if the sphericity assumption was violated. Post-hoc analyses were conducted by means of Fisher’s Least Significant Difference (LSD)-corrected t-tests. In the patient sample, linear correlations (Pearson product-moment correlations) were calculated to assess the relationship between accuracy in all conditions and cumulative fixation duration, as well as accuracy in all conditions, cumulative fixation duration, and the severity of aphasia and apraxia, respectively.

## Results

### 3.1 Accuracy of Speech and Co-Speech Gesture Comprehension

**Fig 3:**
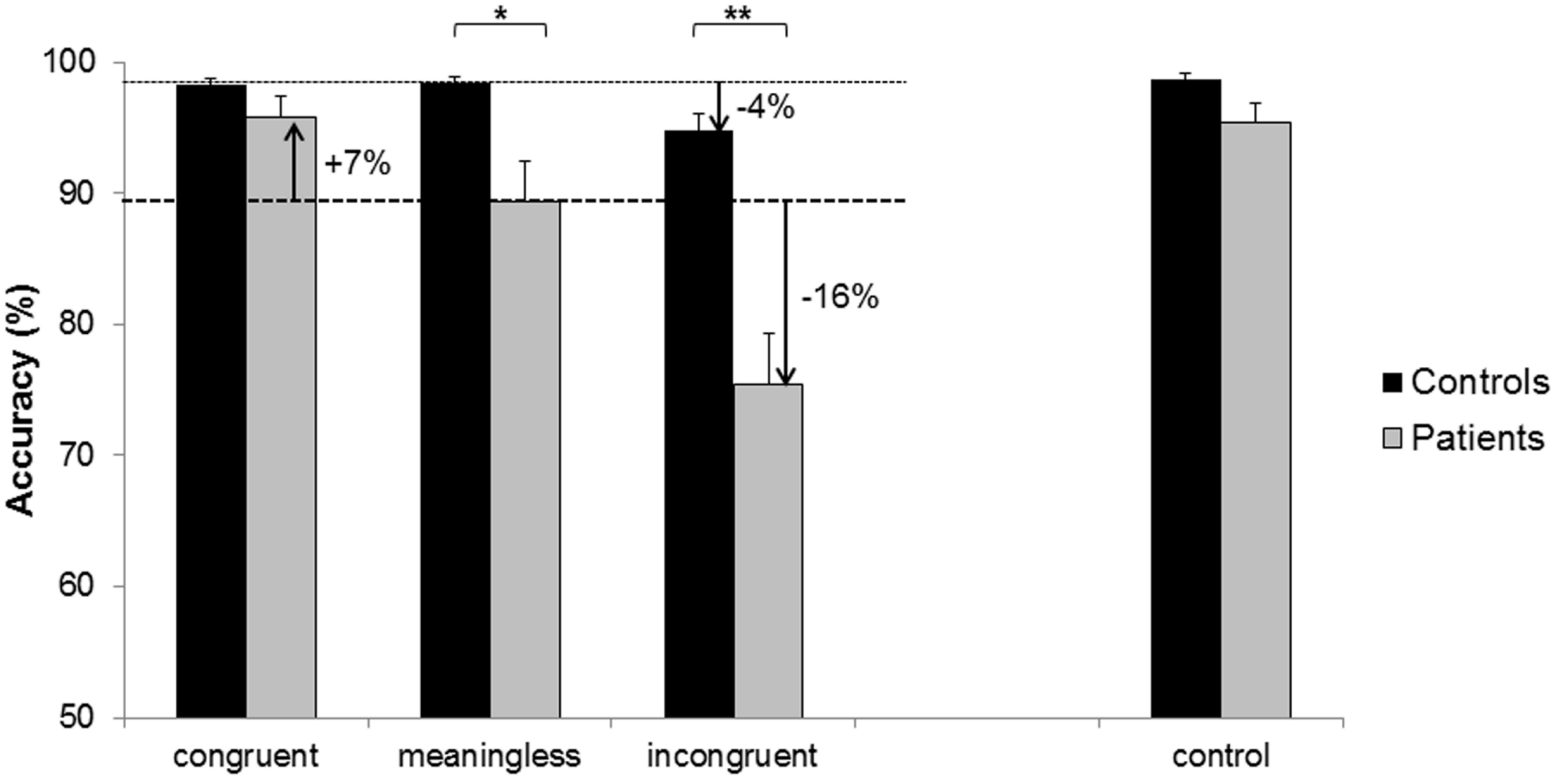
Accuracy for the three conditions of the main experiment, and for the control experiment. The meaningless condition was set as the baseline, and changes in accuracy (i.e., increase or decrease) in the other conditions are indicated by arrows. Asterisks depict significant post-hoc tests (* *p* < .05, ** *p* < .01).

As expected, significant effects on accuracy were found for the between-subjects factor Group (*F*(1, 48) = 21.516, *p* < .01) and for the within-subjects factor Condition (*F*(2, 96) = 40.648, *p* < .01), as shown in Fig 3. Furthermore, the analysis yielded a significant effect of the interaction Group * Condition (*F*(2, 96) = 19.381, *p* < .01). Post-hoc analyses revealed that accuracy did not significantly differ between patients and controls in the congruent condition (patients: *M* = 95.79, *SD* = 7.32, controls: *M* = 98.24, *SD* = 2.67, *p* = .52). However, accuracy was significantly lower in patients than in healthy controls in both the baseline condition (patients: *M* = 89.39, *SD* = 14.20, controls: *M* = 98.35, *SD* = 2.79, *p* = .02) and in the incongruent condition (patients: *M* = 75.45, *SD* = 17.93, controls: *M* = 94.78, *SD* = 6.80, *p* < .01). These results suggest that aphasic patients achieved lower and less consistent overall accuracy scores compared to healthy controls.

Healthy controls performed well across all conditions. Within this group, accuracy was lower for the incongruent condition (*p* = .04), but not significantly different for the congruent condition (*p* = .95) as compared to the baseline condition. In contrast, accuracy within the group of aphasic patients was significantly influenced by the condition. In aphasic patients, accuracy was higher in the congruent condition (*p* < .01), but lower in the incongruent condition (*p* < .01), as compared to the baseline condition.

In the gesturing only control experiment, accuracy was high in both healthy controls and aphasic patients. There was only a small difference in accuracy between the two groups (*F*(1, 22.986) = 11.583, *p* = .05). As a subsequent ANCOVA revealed, accuracy in the control experiment did not explain accuracy in the main experiment (*F*(1,47) = 2.76, *p* = .10). These results and the corresponding post-hoc tests are depicted in Fig 3, including the difference between conditions (i.e., percentage increase or decrease) in the two groups. In patients, accuracy was neither correlated with apraxia nor aphasia severity, for both the main and the control experiment (all correlation coefficients between .01 and .32, with respective p-values between .09 and .47).

### 3.2 Visual Exploration

**Fig 4:**
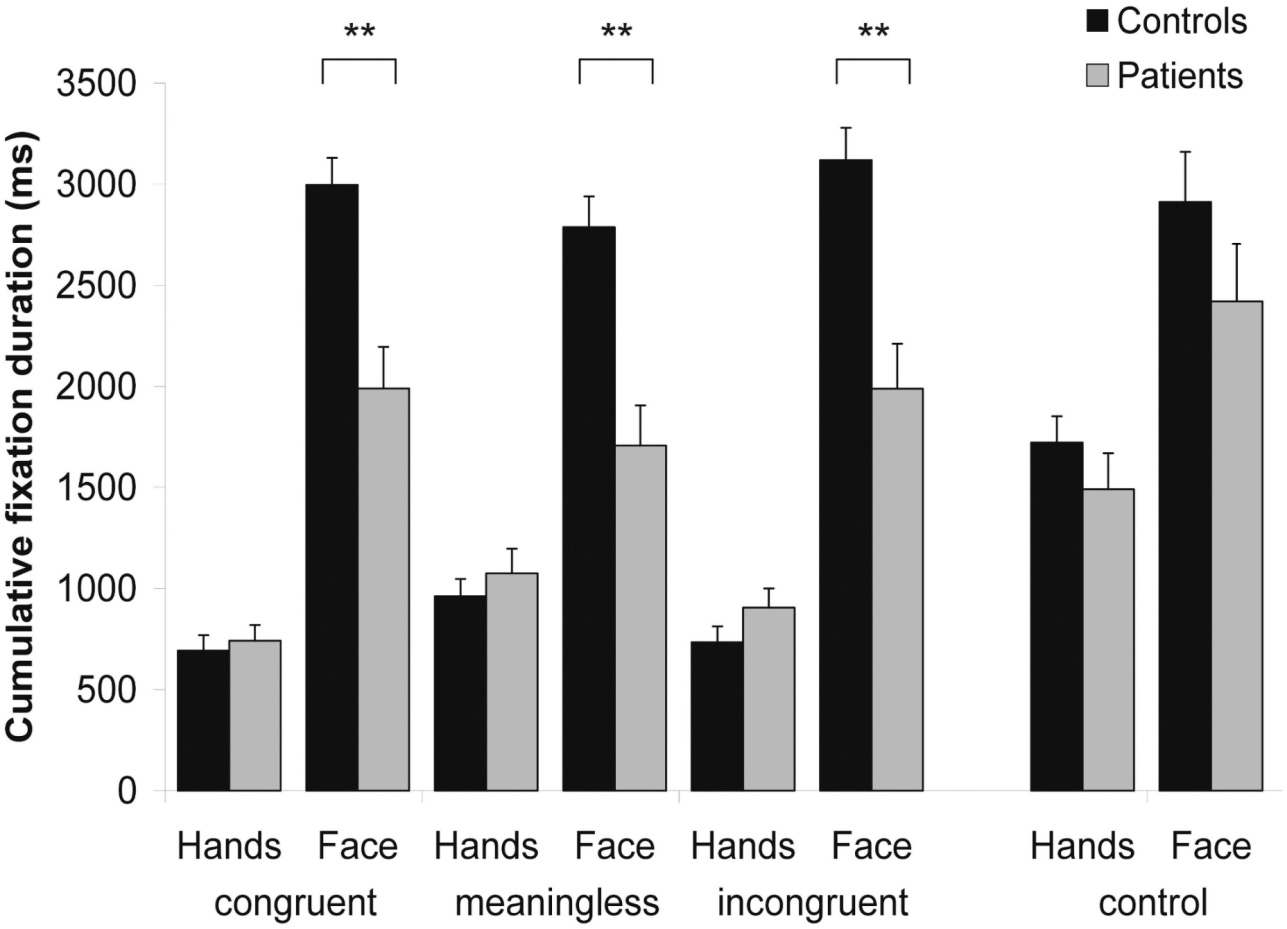
Fixation durations on the regions of interest (ROIs) encompassing face and hands. Values are displayed in milliseconds for the three experimental conditions of the main experiment and for the control experiment, respectively. Asterisks depict significant post-hoc tests (** *p* < .01).

A repeated-measures ANOVA was performed on the cumulative fixation duration data. As shown in Fig 4, significant effects were found for the factors Group (*F*(1, 46) = 21.580, *p* < .01), Condition (*F*(1.771, 81.460) = 9.537, *p* < .01), and ROI (*F*(1, 46) = 85.138, *p* < .01). The analysis further yielded a significant interaction between factors ROI * Group (*F*(1, 46) = 11.956, *p* < .01). Post-hoc analysis revealed that aphasic patients fixated the ROI face for a significantly shorter cumulative duration as compared to healthy controls (*p* < .01).

A significant interaction was also found between factors ROI * Condition (*F*(2, 92) = 51.962, *p* < .01). Post-hoc analysis showed that the ROI face was fixated for a significantly longer cumulative duration in each condition. However, this preference for the ROI face was significantly stronger in the congruent and in the incongruent condition as compared to the baseline condition (*p* < .01 for both comparisons). For the ROI hands, differences in fixation duration were found between the baseline condition and both the congruent and the incongruent conditions (*p* < .01 for both comparisons), but only as a trend between the congruent and the incongruent conditions (*p* = .06). The interactions between factors Group * Condition (*F*(2, 92) = .009, *p* = .99) and ROI * Group * Condition (*F*(2, 92) = 2.137, *p* = .12) were not significant. Taken together, these results suggest that the two groups explored the face and the hands differently. Both groups looked more at the ROI face, but aphasic patients made significantly shorter cumulative fixations on the face compared to the healthy controls. These results and the corresponding post-hoc analyses are depicted in Fig 4.

The analysis of the data of the gesturing only control experiment yielded no differences in cumulative fixation duration between the two groups. The ANOVA revealed a significant effect of factor ROI *(F*(1, 46) = 17.860, *p* < .01), but neither an effect of factor Group (*F*(1, 46) = 2.21, *p* = .14) nor a significant interaction between factors ROI * Group (*F*(1, 46) = .19, *p* = .66).

Correlation analyses revealed significant relationships between aphasia and apraxia severity and visual exploration of the face. Both the scores of the Token Test (as a measure of aphasia severity; *r*(18) = -.43, *p* = .04) and of the TULIA (*r*(18) = .56, *p* = .01) were significantly correlated with the cumulative fixation duration on the ROI face across all three conditions. A trend towards a significant correlation was found between the Written Language Subtest and the cumulative fixation duration on the ROI face (*r*(14) = .43, *p* = .06). Overall, the more severe the aphasia or the apraxia, the less patients looked at the face. In contrast, exploration of the ROI hands was only correlated with the Written Language Subtest scores (*r*(14) = -.56, *p* = .02), but not with the Token Test (*r*(18) = .22, *p* = .19) or the TULIA scores (*r*(18) = -.26, *p* = .14).

In the gesturing only control experiment, neither aphasia (as measured by the Token Test and the Written Language Subtest; *r*(17) = -.24, *p* = .17, and *r*(14) = .37, *p* = .09, respectively) nor apraxia severity (*r*(17) = .08, *p* = .38) were correlated with the cumulative fixation duration on the ROI face. Similarly, the cumulative fixation duration on the ROI hands was neither correlated with the Token Test (*r*(17) = .27, *p* = .14) nor the TULIA scores (*r*(17) = -.18, *p* = .25). However, the Written Language Subtest scores were correlated with the visual exploration of the hands (*r*(14) = -.54, *p* = .02).

No significant association was found between the two main parameters of the present study. In aphasic patients, accuracy and cumulative fixation duration on both ROIs in all three conditions were not significantly correlated (all correlation coefficients between .00 and 0.32, with respective *p*-values between 0.10 and .50).

## Discussion

### 4.1 Accuracy of Speech and Co-Speech Gesture Comprehension

In the present study, we examined the influence of congruence between speech and gesturing on comprehension in terms of accuracy in a decision task in aphasic patients and healthy controls. Our first hypothesis, postulating that accuracy in aphasic patients would be modulated by the level of congruence between speech and concurrent gesturing, was confirmed.

In the baseline condition (i.e., speech combined with meaningless gesturing), aphasic patients showed lower accuracy rates compared to healthy participants. However, in the control experiment testing comprehension of isolated gestures, no significant differences between aphasic patients and healthy controls were found. This suggests that comprehension of isolated gestures per se was not significantly impaired in aphasic patients. This further implies that aphasic patients’ reduced accuracy in the baseline condition is either due to deficits in verbal comprehension or—as we did not specifically measure verbal comprehension alone—general cognitive impairments in brain lesioned patients.

If for instance aphasia-specific comprehension deficits were causing patients’ decreased comprehension in the baseline condition, a correlation between aphasia and / or apraxia severity and patients’ accuracy would be expected, as other studies on aphasia and gesture perception [37, 38] have shown. We did not find such a correlation in our study. This apparent discrepancy to previous research may result from the relatively minor role of praxis for an accurate performance in our task. Furthermore, the co-speech gestures used in the present study were simple and some of our patients were not diagnosed as apraxic or only mildly apraxic. In a study comparing patient groups with differently localized left-hemispheric lesions, it was found that gestural comprehension can be preserved even if gestural imitation is impaired [36].

Thus, further factors such as deficits in memory or executive functions may influence aphasic patients’ comprehension (e.g., [18, 59–62]). As our stimulus material was deliberately simple, short, and explicit, it seems unlikely that memory impairments could have had a major influence on patients’ comprehension. However, it could be that deficits in executive functions play a more important role. Impairments in domains such as reasoning or cognitive flexibility could have interfered with the performance of patients and contribute to the explanation of our findings. Such additional cognitive deficits might thus further explain why there were no significant correlations between aphasia or apraxia severity and patients’ comprehension in terms of accuracy in the decision task.

Congruence between speech and gesturing influenced aphasic patients’ comprehension. In comparison to the baseline condition, congruent co-speech gestures led to increased accuracy, whereas incongruent co-speech gestures led to decreased accuracy. This confirms our first hypothesis that co-speech gestures modulate comprehension in aphasic patients. These effects of congruence or incongruence between speech and gesturing on comprehension are consistent with several previous findings: firstly, incongruent verbal information has been shown to attenuate verbal processing, even in healthy controls (e.g., [31]). In our controls, incongruent co-speech gestures also led to a decrease in accuracy as compared to the baseline condition, although this effect was much less pronounced than in the group of aphasic patients. Secondly, congruent speech and gesturing have been shown to facilitate speech processing. For instance, Marangolo et al. [63] showed that aphasic patients perform better in a verb retrieval task when they previously observed the corresponding action, but they show no improvement when they observed meaningless gestures. The authors postulated that language production is improved by the mere observation of meaningful gestures [55]. This is in line with the concept of multimodal gain, i.e., the beneficial effect of multimodal (auditory and visual) presentation of information. The effect of multimodal gain has been demonstrated in healthy individuals, e.g. in terms of faster speech processing through the presence of gesturing [30]. However, to which extent this effect also holds true for aphasic patients is a matter of debate. Several studies (e.g., [25–29]) have shown that aphasic patients may benefit from information presented in multimodal fashion. In contrast, Cocks et al. [24] found multimodal gain to be significantly impaired in a single case study. Aphasic patients make also significantly more errors in crossmodal matching of visual and auditive information compared to healthy controls [64]. Our results seem to imply that aphasic patients can benefit from multimodal input, but the crucial factor for multimodal gain seems to be congruence between input modalities. A further possible explanation for the difference of our results and the findings of Cocks’ et al. [24] study is the severity of their patient’s impairment; he was diagnosed with a severe Broca’s aphasia.

### 4.2 Visual Exploration

The analysis of participants’ visual exploration behavior during the decision task revealed that both healthy participants and aphasic patients fixated mostly the face region. This finding is in line with our hypothesis that all participants would fixate the face region the most. It is also in line with previous eye-tracking studies (e.g., [39, 42, 43]).

We further hypothesized differences in the visual exploration between aphasic patients and healthy controls. In particular, we hypothesized that they would either pronouncedly fixate the gesturing hands or the face of a person, depending on whether they relied more on nonverbal information due to their language impairments or whether they found gestures to be less informative and comprehensible due to apraxia. We found that aphasic patients fixated the face region less frequently than healthy controls. Aphasic patients seem to allocate more attention (as measured by fixation durations) to the non-verbal information conveyed by the co-speech gestures.

This may be interpreted in terms of a deficit of attentional allocation in the presence of multimodal stimuli (e.g., [65–68]), a theory which is supported by the results of a recent eye-tracking study [69]. Moreover, difficulties in attention allocation seem not to be limited to multimodal stimuli; competing input in the verbal modality alone can already diminish aphasic patients’ comprehension [70]. Alternatively, aphasic patients might have adopted a strategy of trying to avoid the interference presented by multimodal stimuli, regardless of the congruence level and despite possible beneficial effects of multimodal presentation in congruent trials. This might result from their experience throughout the experiment that congruent and incongruent trials were presented randomly and unpredictably. Such a strategy of interference avoidance might also be related to the possibly limited attentional resources as discussed above. However, we did not specifically assess attentional ressources in our patient sample, and can only speculate that our patients also had difficulties to allocate attention efficiently with increasing task demands. The results of our control experiment are also in favor of such an interpretation as no significant differences in terms of visual exploration were found between aphasic patients and healthy controls. This finding implies that the sequential combination of auditory and visual input per se is not crucially relevant, but rather the amount of interference between these two modalities.

In our study, visual attention to co-speech gestures was necessary in order to solve the task. Therefore, we found higher fixation durations on gestures in comparison to previous studies (e.g., [39, 42, 43]). Studies, that applied mismatch paradigms (e.g., [71, 72]), in which verbal input was partially combined with incongruent gestures (e.g., a verbal narration of a cartoon sequence, accompanied by a hand gesture that referred to the wrong character, or a pointing gesture towards the wrong direction, etc.), revealed that gestural stimuli were fixated to a higher extent when they contained information that was necessary in order to solve a task. We could show that this is also the case in our study with aphasic patients.

Interestingly, visual exploration behavior was modulated by the severity of aphasia as well as apraxia: the more severely affected patients were, the less they fixated the face region. The visual exploration behavior of mildly affected patients seemed thus to be similar to the one of healthy controls. Mildly affected patients might be able to allocate their attention more flexibly than severely affected ones, achieving a more efficient exploration strategy.

No significant correlations were found between accuracy in the decision task and visual exploration. This finding is in line with the result of a study by Everdell, Marsh, Yurick, Munhall, and Paré [73], which focused on the investigation of face exploration, also using a paradigm that combined auditory and visual information processing. The authors showed that visual exploration of faces could not predict correct speech perception in healthy participants [73]. It seems that aphasia and apraxia influence visual exploration behavior but do not conclusively determine comprehension in terms of accuracy, suggesting that other factors might play a role.

### 4.3 Conclusions

Our findings ascertain an important role of co-speech gestures for comprehension in aphasic patients. Congruent gestures increase patients’ comprehension in terms of accuracy in a decision task, while incongruent gestures decrease it. The fact that congruent gestures increased comprehension may be a promising approach for clinical and therapeutic purposes. For instance, interaction strategies between therapists and patients could possibly benefit from the deliberate addition of redundant information through co-speech gestures.

Differences between patients and healthy participants were found on the level of visual exploration behavior. In particular, the more severely patients were affected by aphasia as well as apraxia, the more noticeable was the difference in their visual exploration behavior as compared to healthy controls. Aphasic patients might explore the face region to a lesser extent in order to avoid potentially interfering information conveyed by this region. This could be interpreted as a deficit in attention allocation or a strategy of interference avoidance, which, however, proves not to be sufficient to correctly understand speech input. We conclude that the analysis of eye movements is a sensitive method to detect differences in visual exploration of speech and co-speech gesturing between aphasic patients and healthy individuals. However, further research is necessary. For instance, the influence of other cognitive functions such as executive functions or short-term memory may be studied in future studies.

## Supporting Information

S1 Appendix Video Stimuli. (PDF)
