# Supplementary material for: Comprehension of Co-Speech Gestures in Aphasic Patients: An Eye Movement Study
Source: PLoS One. 2016 Jan 6;11(1):e0146583. doi: 10.1371/journal.pone.0146583 (PMC4703302; doi:10.1371/journal.pone.0146583)
Supplement: S1 Appendix — (PDF) [file pone.0146583.s001.pdf]

S1 Appendix 1. Video Stimuli

Main Experiment:

Verbal Stimuli	English	Congruent Meaningful Gesture
**Congruent Condition**		
Bodylotion eincremen	to apply body lotion	applying body lotion
Einen Brief schreiben	to write a letter	writing a letter
Essen schneiden	to cut food	cutting food
Gitarre spielen	to play guitar	playing guitar
Lasso werfen	to throw a lasso	throwing a lasso
Suppe kochen	to cook a soup	cooking a soup
Teig kneten	to knead dough	kneading dough
Wäsche auswringen	to wring out laundry	wringing out laundry
Einen Ball werfen	to throw a ball	throwing a ball
Brille putzen	to clean one's glasses	cleaning one's glasses
Tastatur schreiben	to write on a keyboard	writing on a keyboard
Uhr lesen	to read a watch	reading a watch
Uhr stellen	to set the time on a watch	setting the time on a watch
Baby wiegen	to rock a baby	rocking a baby
Querflöte spielen	to play transverse flute	playing transverse flute
Schirm öffnen	to open an umbrella	opening an umbrella
Bedien' dich ruhig	help yourself	extending open hand, offering something
Ich muss zum Coiffeur	I need a haircut	pointing at one's hair
Meine Schulter ist verspannt	my shoulder is tight	massaging one's shoulder
Aber Achtung	watch out	extending one's index finger
Mach doch eins nach dem andern	make one thing at a time	indicating separate actions with one's hand
Das finde ich nicht so gut	I don't like that	putting one's thumb down
Ganz ruhig	keep calm	moving hands up and down calmly
Gibst Du mir etwas Münz?	can you give me some change?	extending open hand, begging
Mir ist kalt	I am cold	rubbing one's upper arms
Ich habe keine Ahnung	I have no idea	shrugging one's shoulders
Kannst Du das etwas lauter sagen?	can you say that a bit louder?	putting one's hand behind an ear
Es war so lecker	it was so delicious	holding one's stomach as if full
Die Musik ist viel zu laut	the music is much too loud	covering one's ears with both hands
Das gefällt mir	I like this	showing thumbs up
Ich hab Hunger	I am hungry	rubbing one's stomach
Ruf mich doch an	call me	holding one's hand at an ear
Ich habe verloren*	I lost (e.g., a game)	making a fist

Verbal Stimuli	English	Incongruent Meaningful Gesture
**Incongruent Condition**		
Einen Fisch angeln	to angle a fish	cleaning a window
Zähne putzen	to brush one's teeth	dialing a number on a telephone
Mit den Fingern zählen	to count with one's fingers	throwing a dice
Socken stricken	to knit socks	throwing a coin
Zeitung lesen	to read a newspaper	washing one's hands
An eine Türe klopfen	to knock on a door	typing on a keyboard
Blumen giessen	to water flowers	opening a bottle
Ein Getränk einschenken	to pour a drink	reading a newspaper
Nägel kauen	to bite one's fingernails	raising a hand
Eine Flasche öffnen	to open a bottle	Making up one's eyelashes
Einen Schwur leisten	to swear a vow	applying deodorant
Hände waschen	to wash one's hands	knocking on a door
Eine Nummer wählen	to dial a number	swearing a vow
Am Ohr kratzen	to scratch one's ear	fanning oneself
Dieser Kaktus ist aber stachelig	this cactus is thorny	caressing something
Es ist nur ein kleines Sandwich	it's only a small sandwich	mimicking something large

Prost	cheers	eating
Die Herdplatte ist abgekühlt	the cooktop has cooled down	pulling away a hand rapidly
Sie hat jetzt ganz kurze Haare	she has very short hair now	depicting hair of over-shoulder length
Was meinst denn Du dazu?	what do you think about this?	pointing at oneself
Ich bin ganz entspannt	I am totally relaxed	tapping nervously with one's fingers
Das gefällt mir gar nicht	I don't like this at all	thumbs up-sign
Das hast Du gut gemacht	you did a good job	thumbs down-sign
Ich weiss es	I know the answer	shrugging one's shoulders, arms spread out
Ich bin ganz sicher	I am very sure of this	moving a hand upside down

**Verbal Stimuli**	**English**	**Incongruent Meaningful Gesture**
**Baseline Condition**		
Einen Brief öffnen	to open a letter	putting fingers on the contralateral shoulder
Etwas ausradieren*	to erase something	putting both hands flat on the table
In einen Apfel beissen	to bite into an apple	crossing over index and middle fingers
Einen Knopf drücken	to push a button	roll one hand into a fist on the table
Einen Ball rollen	to roll a ball	flip over one flat hand on the table
Eine Briefmarke aufkleben	to put a stamp on a letter	tapping on one's throat with one finger
Eine Brille aufsetzen	to put on glasses	holding one hand perpendicularly to the other
Einen Cocktail mixen	to mix a cocktail	placing thumb on index finger of one hand
Eine Dose öffnen	to open a can	putting one hand on top of the other
Einen Gegenstand abwägen	to weigh an object	tapping one finger on one's cheek
Klavier spielen	to play piano	putting both hands on one's head
Eine Krawatte binden	to knot a tie	knocking on one forearm with one's hand
Einen Kreis zeichnen	to draw a circle	pulling one's earlobe with two fingers
Ein Messer schleifen	to sharpen a knife	stretching out one's elbow
Ein Streichholz anzünden	to light a match	putting both thumbs on the middle fingers
Ein Tablett halten	to hold a tray	holding both fists in front of one's upper body
Einen Tisch abwischen	to wipe a table	tapping on one's head with a hand

Note. * = omitted from analysis

**Control Experiment: Performed Gesture**	**English**	**Choice of Possible Answers (Correct Answer in Bold)**
Auto fahren	to ride a car	to ride a motorcycle / **to ride a car** / to wash a car
Nägel lackieren	to paint one's nails	**to paint one's nails** / to cut one's nails / to paint a car
Hände dehnen	to stretch one's hands	to clap one's hands / **to stretch one's hands** / to stretch one's legs
Baby wiegen	to rock a baby	to measure flour / to change a baby / **to rock a baby**
Hände waschen	to wash one's hands	**to wash one's hands** / to stretch one's hands / to do laundry
Zeitung lesen	to read a paper	to deliver a paper / **to read a paper** / to read a book
Tastatur schreiben	to write on a keyboard	**to write on a keyboard** / to fix a keyboard / to write a letter
Essen schneiden	to cut food	to cut a paper / **to cut food** / to cook
Uhr lesen	to read a watch	to read a paper / to set a watch / **to read a watch**
Klavier spielen	to play the piano	**to play the piano** / to tune a piano / to play cards
Komm mal näher	come on closer	move over / **come closer** / come for dinner
Aber Achtung	watch out	stop / nothing is going to happen / **watch out**
Es ist nur rein kleines Sandwich	it's only a small sandwich	**it's only a small sandwich** / it's only a little child / it's a big sandwich
Kannst Du das etwas lauter sagen?	can you say that a bit louder?	my ear is itchy / **can you say that a bit louder?** / the music is much too loud
Sie hat jetzt ganz kurze Haare	she has very short hair now	she has long hair now / **she has very short hair now** / she is wearing a short skirt today
